# Thiol–Ene Cross-linking of Poly(ethylene glycol) within High Internal Phase Emulsions: Degradable Hydrophilic PolyHIPEs for Controlled Drug Release

**DOI:** 10.1021/acs.macromol.1c01240

**Published:** 2021-11-08

**Authors:** Viola Hobiger, Anna Zahoranova, Stefan Baudis, Robert Liska, Peter Krajnc

**Affiliations:** † PolyOrgLab, Faculty of Chemistry and Chemical Engineering, University of Maribor, Smetanova 17, Maribor 2000, Slovenia; ‡ Institute of Applied Synthetic Chemistry, Vienna University of Technology, Getreidemarkt 9/163, Vienna 1060, Austria

## Abstract

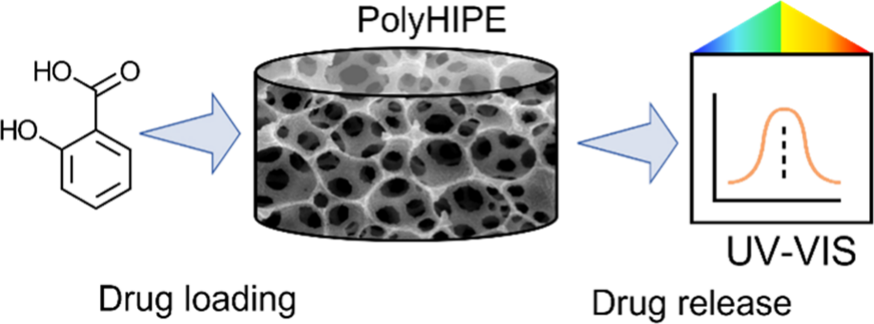

Macroporous polymer monoliths prepared from high internal phase emulsions (HIPEs) can be found in various biomedical applications. While typically water-in-oil HIPEs are applied for polyHIPE preparation, they are not suitable for hydrophilic polyHIPE preparation. Herein, direct oil-in-water emulsions based on water-soluble poly(ethylene glycol)diacrylate or poly(ethylene glycol)dimethacrylate were developed. Furthermore, the incorporation of a hydrophilic water-miscible thiol, ethoxylated trimethylolpropane tris(3-mercaptopropionate) (ETTMP) was reported for the first time within thiol–ene polyHIPEs. Due to the transparency of the emulsions, rapid curing via photopolymerization was feasible. The average pore diameters of the resulting polyHIPEs ranged between 1.2 and 3.6 μm, and porosity of up to 90% was achieved. The water uptake of the materials reached up to 1000% by weight. Drug loading and release were demonstrated, employing salicylic acid as a model drug. Porous profile and biodegradability add to the usefulness of the material for biomedical applications.

## Introduction

Research on porous polymers has expanded into different directions, such as gas and energy storage, separation methods, heterogeneous catalysis,^1^ and the biomedical sector.^2^ Cross-linked polymers with micrometer-sized pores and an interconnected open porous structure are especially interesting for biomedical applications. They can be employed for tissue engineering, tissue regeneration, cell culturing, wound dressings, or controlled release.^2^ One of the routes leading to such macroporous polymers is the emulsion templating method utilizing high internal phase emulsions (HIPEs). The internal phase volume in a HIPE reaches above 74%, resulting in a polyhedral shape of the dispersed droplets and only a thin layer of continuous phase coating them. Upon polymerization of the continuous phase, an interconnected macroporous polymer, a so-called polyHIPE, is obtained. One of the advantages of emulsion templating is that the morphology can be tuned on several levels, ranging from overall porosity to pore size to the interconnectivity of pores.^3^ The ability to tweak material properties and the sheer scope of different chemistries available are the reason why polyHIPEs have become an increasingly recognized field of research.^4^ The potential of polyHIPEs within the area of biomedical applications, where precautions to prevent toxicity and *in vivo* incompatibility must be taken, is underlined by a recent review on the matter.^5^

The majority of research on polyHIPEs focuses on hydrophobic systems through water-in-oil (w/o) emulsions. However, when a biomedical application is envisioned, a hydrophilic material is often more suitable. To achieve hydrophilic materials, a different approach for synthesis is needed. Lee et al. reported a method to produce hydrogels from poly(vinyl alcohol) by concentrated CO_2_-in-water (c/w) emulsions.^6,7^ Poly(acrylamide)s for tissue engineering were also created using a poly(vinyl alcohol)-based c/w HIPE precursor.^8^ Another approach made use of post-polymerization processing like hydrolysis.^9^ The group of Krajnc et al. has successfully applied different techniques to achieve hydrophilic polyHIPEs, ranging from direct “reversed” oil-in-water (o/w) acrylic acid emulsion to polyHIPEs based on different acrylates, such as 2-hydroxyethyl methacrylate (HEMA) and ethylene glycol dimethacrylate (EGDMA).^10,11^ A similar approach by Nalawade et al. was termed as “inverse” high internal phase emulsions. It featured porous materials from glycerol monomethacrylate, HEMA, and glycerol dimethacrylate (GDMA) for tissue engineering purposes.^12^ Recently, Golub also published a procedure leading to an *N*,*N*′-methylenebisacrylamide cross-linked HEMA polyHIPE for dye absorption.^13^

For biomedical applications, naturally derived polymers like gelatin, dextran, or alginate have been used as well.^14−16^ Their advantage is the biocompatibility as well as their degradability. They, however, tend to have worse mechanical properties compared to synthetic alternatives. One of the synthetic building blocks found in polyHIPEs for biomedical applications is poly(ethylene glycol)-based monomers. The group of Wynne developed a protocol for emulsion-templated poly(ethylene glycol)diacrylate (PEGDA) and sodium/calcium polyacrylate and PNIPAM polyHIPEs for wound dressings.^17^ Also, poly(ethylene glycol)methacrylate (PEGMA) has been added to photopolymerized HIPE formulations by Kimmins et al. to increase general hydrophilicity.^18^ Polyacrylate-PEG biomaterials for drug release have also been demonstrated by Corti et al.^19^

The aim of this study was to create a simple one-pot procedure including PEG-based monomers with a fast-curing mechanism via photo-induced thiol–ene click chemistry. Thiol–ene click polymerization has become increasingly popular for the synthesis of polyHIPEs since first reported by Lovelady et al. due to its easy use and potential for biomedical applications.^20,21^ One of the reasons why thiol–ene polyHIPEs are suitable for biomedical applications is their degradabability due to the hydrolysis of ester linkages. Degradable thiol–ene polyHIPE scaffolds for tissue engineering were first reported by Caldwell et al. with a network based on trimethylolpropane tris(3-mercaptopropionate) (TMPMP), trimethylolpropane triacrylate (TMPTA) and dipen-taerythritol penta/hexa-acrylate. They investigated degradation under accelerated and cell culture conditions.^22^ The advantage of less oxygen inhibition of the thiol–ene reaction was also shown by the group of Cosgriff-Hernandez.^23^ To this day, a very limited number of thiol cross-linkers has been applied, namely, TMPMP and pentaerythritol tetrakis(3-mercaptopropionate).

**Figure 1:**
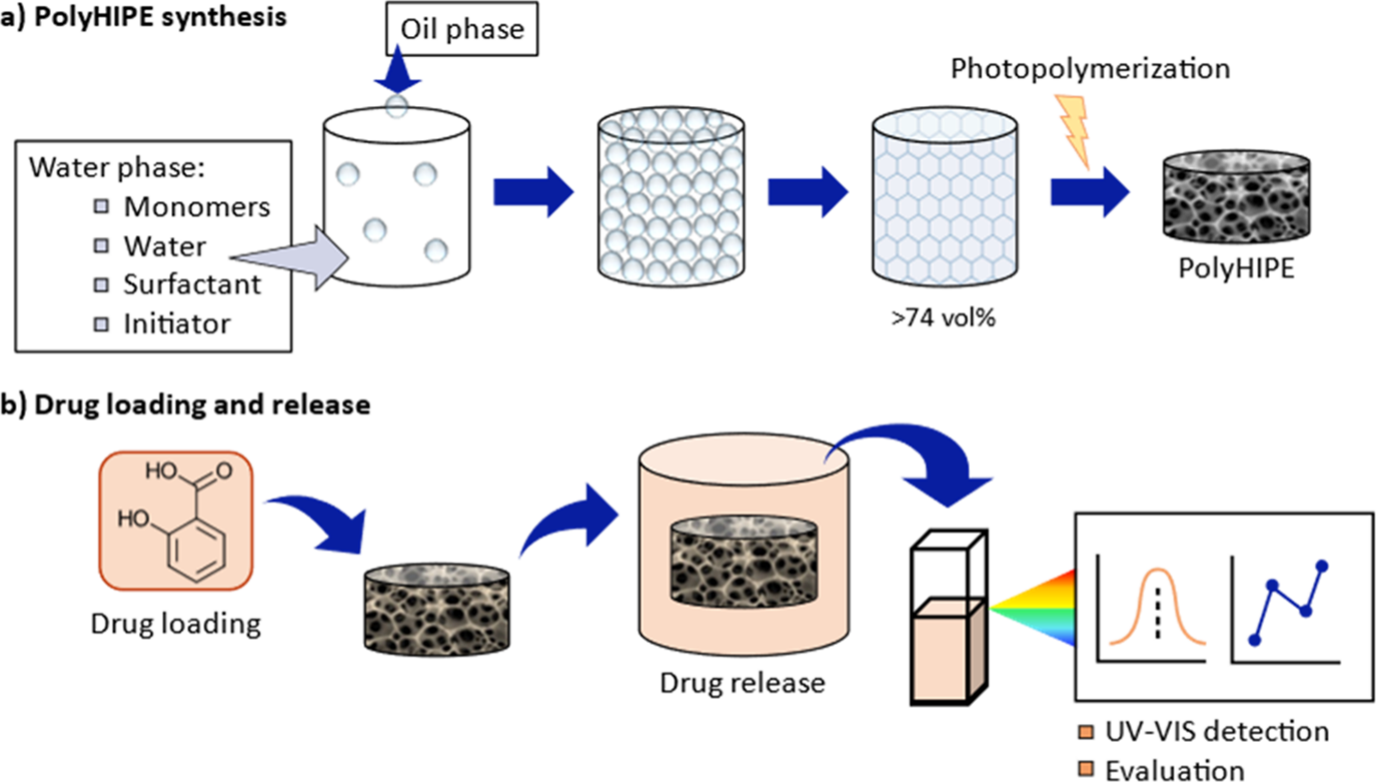
Depiction of the two main aspects of the present work, with (a) synthesis and development process of our materials via a high internal phase emulsion and (b) loading of salicylic acid and the release process observed over time via UV/vis spectroscopy.

While the examples mentioned above of thiol–ene polymerizations are applied in w/o emulsions and led to rather hydrophobic polymer networks, the focus of this work lay on the direct high internal phase emulsions, applying water-miscible monomers to prepare hydrophilic, degradable porous materials with open cellular morphology. For the first time, a stable o/w HIPE, directly incorporating a hydrophilic thiol, was developed. A PEG-based system was chosen due to being water-soluble, non-toxic, and reportedly biocompatible. These systems find widespread use and recognition in biomedical applications like drug delivery and tissue engineering and can be found in combination with thiol-cross-linkers.^24−26^ Photopolymerization was chosen as a fast-curing mechanism to achieve thiol–ene hydrogel polyHIPEs. Foreseeable applications of such porous polymers lie in tissue regeneration, controlled release, and water remediation. The impact of various formulation parameters was monitored on structural characteristics, as well as mechanical, water uptake, and swelling behavior of the resulting materials. Furthermore, selected samples were loaded with a model drug, salicylic acid, to demonstrate the potential of our material for sustained drug delivery (see Figure 1).

## Experimental Section

### Materials

The monomers poly(ethylene glycol)dimethacrylate (PEGDMA, average *M*_n_ 750, Sigma-Aldrich), PEGDA (average *M*_n_ 575, Sigma-Aldrich), and ethoxylated trimethylolpropane tri(3-mercaptopropionate) (Thiocure ETTMP 1300, *M*_n_ 1300, Bruno Bock) were all used without further purification. HEMA (Sigma-Aldrich) was passed through Al_2_O_3_ prior to use. Cyclohexane (Carlo Erba Reagents), 2-propanol (Carlo Erba Reagents), ethanol (Australco), polyoxyethylene-polyoxypropylene block copolymer (Pluronic F-68, Sigma-Aldrich), lithium phenyl-2,4,6-trimethylbenzoylphosphinate (Li-TPO, a gift from TU Wien and prepared according to the procedure described in the work of Benedikt et al.),^27^ salicylic acid (99.5%, J. T. Baker), sodium chloride (NaCl, Carl Roth), potassium chloride (KCl, Merck), disodium phosphate (Na_2_HPO_4,_ Sigma-Aldrich), monopotassium phosphate (KH_2_PO_4,_ Merck), sodium acetate (Sigma-Aldrich), and acetic acid (Carl Roth) were used as received.

### General Procedure for the Preparation of PEGDA or PEGDMA PolyHIPE

PEGDMA (2.25 g, 3 mmol) or PEGDA (2.3 g, 4 mmol), Pluronic F-68 (15 wt % of monomers), Li-TPO (1 wt % of monomers), and deionized water (1.5 mL) with CaCl_2_ (0.884 g/100 mL) were added to a brown two-necked round-bottom flask. The flask was secured to an overhead stirrer equipped with a D-shaped blade and left to stir at 300 rpm until a homogeneous mixture was achieved. Then, cyclohexane was added dropwise to the mixture to form a stable HIPE. The HIPE was left stirring for 30 min after the addition of cyclohexane (10.6 mL) was completed. It was transferred to a silicone mold and cured in a UV-chamber (Uvitron Intelli-ray 600, halide lamps, 600 W, 120 mW cm^–2^, 320–580 nm) for 60 s at 70% intensity (61.8 mW cm^–2^). The resulting monolith was washed immediately with 2-propanol and subsequently cleaned by Soxhlet extraction with 2-propanol for 10 h. The cleaned monolith was left to dry slowly under air in the fume hood, followed by drying in a vacuum oven at 35 °C for 16 h.

### Nomenclature of Prepared Samples

Due to the amount of different prepared specimens, a simple nomenclature was established. Samples are either labeled as DA or DMA, depending on the main component. For those named only (PEG)DA_# or (PEG)DMA_#, the _# (e.g., 80) indicates the added internal phase by volume. For samples containing _#T and/or _#H (e.g., 5), T represents thiol, H represents HEMA, and the number (#) represents the amount of the added monomer compared to acrylate or methacrylate in mol %.

### Characterization

The internal morphology of the prepared porous monoliths was examined by scanning electron microscopy (SEM) on different scanning electron microscopes, Quanta FEI 200 3D (FEI Company, United States) operating at 15 kV or SIRION FEI NC 400 (Philips, Netherlands) operated at 10 kV, where samples were gold-coated using a JEOL JFC-1100E (JEOL, Japan) sputtering system for 25 s at 10 mA. Furthermore, an FEI Quanta 200 MK (FEI Company, United States) and a JEOL JCM-6000 (JEOL, Japan) were used, with which samples were gold-coated with an Agar Sputter coater B7340 system for 25 s at 10 mA. The average pore diameters of the cavities were determined by computing the average of 50 pore measurements of a sample with a correction factor of 2/√3. Pore diameters were measured manually by the open-source imaging processing software ImageJ from the National Institutes of Health, USA. Thermal properties, namely, the glass transition temperature, of the material were investigated via dynamic scanning calorimetry with a Mettler Toledo DSC 3 equipped with STAR^e^ software (Mettler Toledo, United States). Samples between 2 and 9 mg were weighed and heated at a rate of 20 °C/min from room temperature to 100 °C and then cooled to −70 °C at a rate of 10 °C/min. The temperature was kept at −70 °C for 10 min and was then raised to 100 °C at a rate of 5 °C/min. Glass transition temperature was determined from the resulting thermographs of each sample (*n* = 2).

The skeletal density ρs of the materials was determined using a Micromeritics Accupyc II 1340 helium gas pycnometer. Envelope density was measured by densitometer Micromeritics GeoPyc 1365. The porosity of the samples was calculated as follows
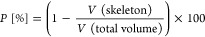
1

Theoretical porosity was calculated from the deployed water phase and solvent. Nitrogen adsorption/desorption measurements were performed on a Micromeritics TriStar II 3020 porosimeter using the Brunnauer Emmet Teller model for the evaluation of the surface area. Refractometry was measured with a Mettler Toledo RE40 Refractometer.

### Gel Content

An aliquot of the emulsion was taken and weighed prior to polymerization. After polymerization, the polymer aliquot was cleaned with 2-propanol in a Soxhlet apparatus for 5 h. Afterward, 2-propanol was replaced with water and repeatedly exchanged over 24 h. The swollen specimen was freeze-dried, and the dry weight was noted. The gel content was calculated as a ratio of the weight of the washed and dried sample to the theoretical polymer weight calculated from the feed.

### Material Properties

Specimen for tensile tests were prepared by casting the HIPE formulation into a transparent silicone mold in dog-bone shape for tensile tests with 12 mm gauge length. The formulations were prepared and treated according to the general synthesis procedure described above. All samples were conditioned in water for 24 h prior to testing. Tensile tests were performed on a Zwick Z050 machine equipped with a 1 kN sensor. The strain was measured with a mechanical extensometer. Results were recorded and analyzed by testXpert II testing software. The crosshead speed was set for 10 mm·min^–1^. At least, three specimens were tested for every formulation.

### Water Uptake Studies

The washed and dried polyHIPE samples were broken into pieces of approximately the same size and weight of around 30 mg. To every sample, 2 mL of prepared PBS (phosphate-buffered saline) solution (pH = 7.4) was added to the closed container and kept at room temperature. Triplicate analysis was performed in all cases. Excess buffer was removed with a filter paper before the weight of the samples was recorded after 15 min, 1 h, 24 h, and 96 h. The water uptake was calculated from the average weight after 24 and 96 h combined. The uptake percentage (WU) was calculated as follows with *W*_s_ (weight swelled) and *W*_d_ (weight dry)
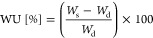
2

### Degradation Studies

The washed and dried polyHIPE samples were broken into pieces of approximately the same size and weight of around 10 mg. Triplicate analysis was performed in all cases. To every sample, 2 mL of either PBS buffer solution (pH = 7.4) or acetate buffer solution (pH = 4.9) were added, and the container was closed and stored in an oven at 37 °C without shaking. After 1, 2, 3, and 6 weeks, samples were withdrawn from the solution, washed three times with deionized water, and placed into a vacuum oven for 48 h. Afterward, the dried samples were weighed, and the weight change was documented. For the remaining samples continuing incubation, the pH of the solution was recorded before being exchanged for fresh buffer solution. The remaining mass (RM) from the initial sample weight was calculated as follows from the dry weight (*W*_d_) before degradation and the dry weight after degradation and washing (*W*_da_)
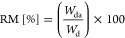
3

### Drug Loading

Three different polyHIPEs were chosen for drug loading and release experiments, namely, DA_5T, DA_10T_10H, and DMA_10T_10H. All samples were washed, dried, and prepared in triplicates. Samples were cut in approximately the same rectangular shape of 0.5 × 1 × 0.15 cm and weighed. The dry weight was recorded, and the samples were immersed separately in an ethanol solution of salicylic acid with a concentration of 100 mg/mL for 24 h. The amount of added solution for each sample was 1 mL per 10 mg sample. After 24 h, the samples were removed from the solutions, and excess liquid was removed by blotting on filter paper. The loaded polyHIPEs were dried in a vacuum oven at 35 °C overnight. The dried specimens were weighed again, and the weight change was recorded. Drug loading was recorded as loaded drug in grams per gram polyHIPE.

### Drug Release

The release was performed with the previously loaded polyHIPEs. Samples were immersed in 10 mL of PBS and incubated at room temperature without agitation. The buffer was exchanged for fresh solution after 5 min, 10 min, 15 min, 30 min, 60 min, 120 min, and 24 h and 48 h. The absorption of the collected samples was measured with UV/Vis spectrometry at a wavelength of 297 nm. A UV-1800 instrument (Shimadzu, Kyoto, Japan) equipped with a six-cell thermoelectrical temperature controller CPS-240A (Shimadzu, Kyoto Japan) was used for measuring the UV/vis spectra. The concentration and cumulative release over time of the discharged drug were calculated using a prepared calibration curve. The calibration curve was prepared from a stock solution of 100 μg/mL of salicylic acid in PBS buffer. Concentrations considered for the calibrations curve were 5, 10, 20, 30, 40, 50, and 60 μg/mL. The drug release profiles were reported as percentage cumulative release respect to the amount of loaded drug (considered as 100%).

## Results and Discussion

### Synthesis of PEG-Based PolyHIPEs Cross-linked by Thiol–Ene Reaction

Several initial factors were evaluated for the o/w polyHIPEs, the most important one being the appropriate surfactant system. A guide for choosing a suitable non-ionic surfactant is the hydrophilic–lipophilic-balance value (HLB), where low HLB corresponds to a molecule with a high proportion of lipophilic groups and vice versa. For this type of emulsion, surfactants with a high HLB-value are desired.^28^ Three block copolymer type surfactants, namely, Pluronic F-68 (HLB = 29), Pluronic F-127 (HLB = 18–23), and Triton X-705 (70%) (HLB = 18.4) were selected based on our previous experience with o/w high internal phase emulsions. It was possible to produce stable emulsions with each of the chosen surfactants, but the typical interconnected pore structure of the resulting polyHIPE was only achieved with Pluronic F-68. This surfactant was thus selected for further studies.

The positioning of the initiator within the polymerization system significantly affects the morphology of the resulting polyHIPE. It was shown in previous research that an initiator, solely distributed in the continuous phase, produced interconnected pores.^29−33^ In contrast, if the locus of initiation was at the interface of the two phases, closed pore structures were observed.^34^ Robinson et al. showed that initiation in the aqueous phase of a w/o HIPE leads to a closed pore architecture.^35^ The water-soluble Li-TPO was chosen as the initiator due to the high efficiency and solubility compared to commonly used Irgacure 2959.^27^ Also, water was added to the continuous phase as a solvent for the initiator and monomers and enhanced the overall emulsion stability. Diffusion of the initiator into the internal, organic phase would be limited, and an interconnected structure was expected.

PolyHIPE samples with varying parameters were prepared (see Table 1). First, the formulations containing only PEG-based monomers were examined. The internal phase volume that could be added without destabilization of the HIPE was studied. For PEGDA, a maximum internal phase volume of 85% was reached before emulsion destabilization. Only 80 vol % of the internal phase could be added in the case of PEGDMA without destabilization. The divergence between the stability of two systems probably results from the different viscosities of the PEG-monomers, leading to a more stable emulsion at higher internal phase volume for PEGDA-based HIPEs. The resulting polymers based on PEGDA and PEGDMA were rather brittle and unsuitable for hands-on application, for example, wound dressing.

**Table 1:** Experimental Conditions for the Different PEG-Based HIPE Formulations

	continuous phase						internal phase
sample	PEGDA [g]	PEGDMA [g]	Thiol [g]	HEMA [g]	Pluronic F-68 [g]	H_2_O [mL]	cyclohexane [mL]
DA_75	2.3				0.342	1.5	10.7
DA_80	2.3				0.342	1.5	15.2
DA_85	2.3				0.342	1.5	20.1
DA_5T	2.3		0.26		0.384	1.5	11.7
DA_15T	2.3		0.78		0.462	1.9	14
DA_10H	2.3			0.05	0.352	1.5	11.6
DA_40H	2.3			0.2	0.345	1.5	11
DA_5T_5H	2.88		0.325	0.035	0.485	2.2	15.9
DA_10T_10H	2.88		0.65	0.07	0.539	2.3	16.6
DMA_75		2.25			0.337	1.5	10.6
DMA_80		2.25			0.337	1.5	14.2
DMA_5T		2.25	0.195		0.245	1.5	11.1
DMA_10H		2.25		0.04	0.344	1.5	11.4
DMA_20H		2.25		0.08	0.344	1.5	11.1
DMA_5T_5H		3	0.26	0.026	0.493	2.1	15.2
DMA_10T_10H		3	0.52	0.052	0.536	2.3	16.4

**Figure 2:**
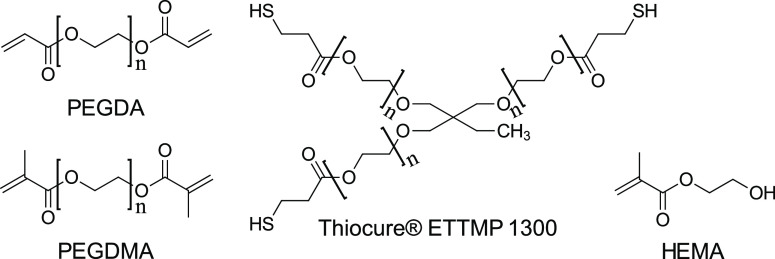
Monomers used for polyHIPE synthesis.

The trifunctional ETTMP 1300 was added with the intent of creating mechanically more stable materials and increasing degradability via the introduction of more ester linkages to the system (see chemical structure in Figure 2). Thiol addition leads to a mixed polymerization mechanism of chain growth and step growth, creating a more uniform network and significantly increasing toughness.^36^ Thiyl radicals are also less sensitive to oxygen inhibition, and the gel point is reached later compared to a chain-growth mechanism. The mono-functional monomer HEMA was introduced to the system as well in hopes of increasing the tensile strength.

**Figure 3:**
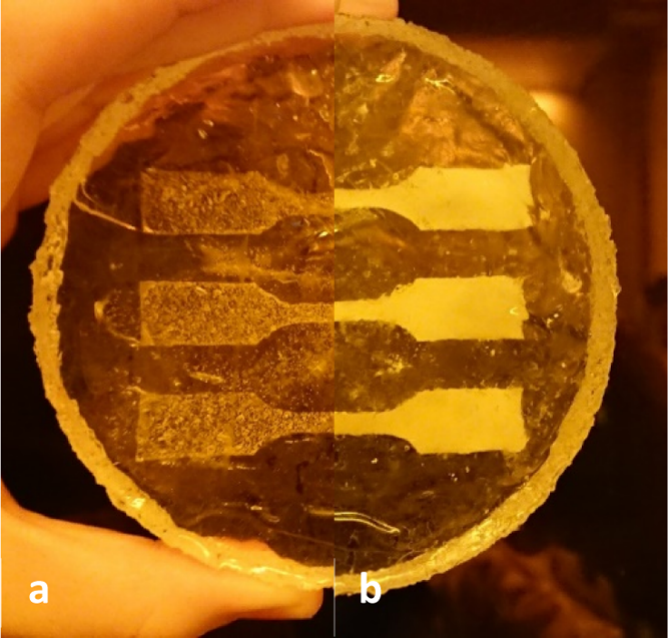
PEGDMA-HIPE containing a silicone mold prior to polymerization (a) and the resulting, opaque PEGDMA–polyHIPE after the polymerization (b).

During our experiments, an unexpected observation was made prior to polymerization of the emulsion. Usually, high internal phase emulsions are opaque-white due to light scattering. However, our produced HIPEs from cyclohexane and an aqueous monomer phase were transparent (see Figure 3a). There are two possible explanations for the transparency of the emulsions. The phenomenon could be the consequence of the emulsion droplets being smaller than the wavelength of the penetrating light or the two phases being isorefractive. Scanning electron micrographs of the resulting polyHIPEs showed average cavity diameters of 2.2 μm, suggesting emulsion droplets larger than the borderline for light scattering (see Table 2). Refractive index measurements for cyclohexane and PEG-formulations revealed differences in the refractive indices between 0.001 and 0.0047 (see Table 2). Transparent emulsions were reported for similar values suggesting that the transparency of the herein produced HIPEs results from isorefractive phases.^37^

**Table 2:** Structural Properties of the Different Prepared PolyHIPE Samples

sample	cavity diameter^a^ [μm]	theoretical porosity [%]	porosity^b^ [%]	gel content [%]	RI difference^c^
DA_75	2.25 ± 0.65	83	88	>99	0.0017
DA_80	2.12 ± 0.54	88	90		
DA_85	2.08 ± 0.58	90	89		
DA_5T	3.06 ± 0.67	83	86	83	0.0047
DA_15T	1.19 ± 0.22	83	61	88	0.0028
DA_10H	2.65 ± 0.46	84	86	89	0.0026
DA_40H	3.58 ± 0.61	82	86	80	0.0046
DA_5T_5H	2.28 ± 0.42	84	86	84	0.0024
DA_10T_10H	2.21 ± 0.41	83	73	86	0.0023
DMA_75	1.95 ± 0.31	83	85	>99	0.0022
DMA_80	1.64 ± 0.33	87	90		
DMA_5T	1.57 ± 0.29	83	77	84	0.0044
DMA_10H	1.82 ± 0.28	84	82	78	0.0032
DMA_20H	2.07 ± 0.39	83	77	78	0.0012
DMA_5T_5H	2.09 ± 0.31	83	75	83	0.001
DMA_10T_10H	2.41 ± 0.54	83	48	86	0.0018

a Average cavity diameter of 50 pores.

b Calculated as stated in the Experimental Section.

c Measured at 25 °C, values were determined by comparing RI of cyclohexane with the RI of the respective water phase prior polymerization without the initiator.

**Figure 4:**
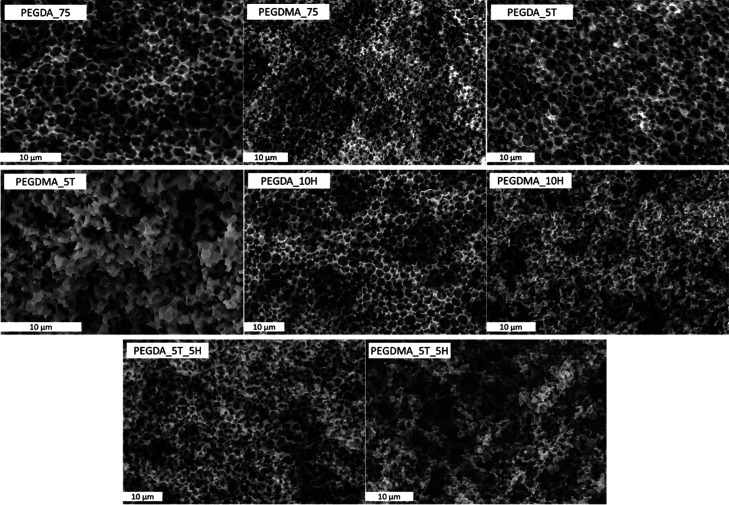
SEM micrographs of selected PEDGA and PEGDMA polyHIPE samples.

Upon drying of the polyHIPEs, shrinkage of some monoliths was observed, primarily for the ones with a mixed monomer composition, including DA_15T, DMA_5T, DA_5T_5H, DA_10T_10H, DMA_5T_5H, and DMA_10T_10H. One reason for this phenomenon could be the negative pressure arising from solvent evaporation. Since those samples exhibit lower interconnectivity based on SEM observations (see Figure 4). The phenomenon of negative pressure could lead to collapse of the interconnected porous structure. However, extensive shrinkage could be prevented by immediate washing with 2-propanol and later exchanging the solvent for water and freeze-drying the samples. SEM micrographs showed that the porous morphology stayed intact for all the investigated samples.

Overall, the developed procedure proved to be very versatile. This was demonstrated by the synthesis of a wide range of different polyHIPEs with different chemical compositions. The unexpected transparency of the emulsion enabled rapid curing times of 60 s or less and high curing depths. Curing depth was investigated within transparent vessels of different diameters. The largest investigated diameter was 27.52 mm (see Supporting Information).

### Material Characterization

After polyHIPE synthesis, the materials based on different formulations were characterized. Average pore sizes were relatively small and determined from SEM images. The range of pore size for polyHIPEs usually lies above 1 μm.^38^ For our materials, the determined diameters ranged from 1.19 μm for DA_15T to 3.58 μm for DA_40H (see Table 2). High porosities were determined for all samples; however, lower porosity was observed with an increased thiol-cross-linker content. This could be especially observed for samples DA_15T, DA_10T_10H, and DMA_10T_10H (Table 2). An increased amount of internal phase volume also led to higher surface area in the case of DA_75, DA_80, and DA_85 (see Supporting Information).

Generally, all developed formulations showed porous morphology, as can be seen in Figure 4. However, some differences could be observed depending on the monomer composition. The intended interconnected porous morphology of PEGDA-based monoliths was less influenced by adding thiol or HEMA compared to PEGDMA. Monoliths with higher amounts of added thiol, like DA_15T and DA_10T_10H, appear to have a less interconnected pore structure, as shown in the recorded SEM images. When only HEMA was added to a PEGDA-based formulation, no changes in the open porous morphology were observed.

Gel content was assessed, and illustrated monoliths consisting solely of acrylate or methacrylate showed full conversion. For the other formulations, a gel content between 80 and 90% was determined, as shown in Table 2. Only DMA_10H and DMA_20H exhibited a gel content of 78%. The low gel content could be due to the homopolymerization of HEMA, resulting in the exclusion from the network and being washed out. The low gel content for DA_40H could either result from homopolymerization or HEMA reacting faster than the acrylate and leaving unreacted PEGDA to be washed out.^39^

In general, for samples based on PEGDA, the addition of thiol decreased brittleness compared to samples without added thiol. It improved the tensile strength and elongation at break of the material, making it more suitable for handling in an application (see Table 3). In comparison with the results obtained by Corti et al., the measured tensile strength was lower. With their polyacrylate/PEG materials, up to 0.91 MPa of tensile strength was achieved compared to 0.62 MPa of the herein reported thiol/PEG materials. However, significantly higher elongation at break could be reached in our case.^19^ Adding just HEMA to PEGDA formulations had almost no effect on improving elongation at break; however, the calculated Young’s modulus decreased less compared to PEGDA-samples containing thiols (see Table 3). Material properties of PEGDMA formulations with HEMA led to decreased tensile strength and elongation at break compared to DMA_75. The addition of thiol to the PEGDMA formulation led to a higher Young’s modulus, but decreased elongation at break was observed. Finally, a combination of PEG-*co*-ETTMP–HEMA–polyHIPEs was created to combine all the envisioned properties.

**Table 3:** Results of Mechanical and Thermal Analysis of Prepared Specimen

sample	Young’s modulus [MPa]	tensile strength *F*_max_ [MPa]	elongation at break [ %]	glass transition temperature [°C]
DA_75	0.46	0.07 ± 0.02	16.0 ± 4.66	–41.23
DA_5T	0.31	0.11 ± 0.01	36.6 ± 3.60	–36.54
DA_15T	0.25	0.14 ± 0.00	55.0 ± 1.77	–42.27
DA_10H	0.43	0.08 ± 0.02	18.2 ± 3.53	–32.26
DA_40H	0.47	0.09 ± 0.01	18.9 ± 2.49	–26.40
DA_5T_5H	0.26	0.12 ± 0.01	47.3 ± 4.89	–36.64
DA_10T_10H	0.22	0.09 ± 0.03	42.7 ± 18.86	–39.72
DMA_75	0.26	0.11 ± 0.02	44.4 ± 9.03	–43.52
DMA_5T	0.40	0.14 ± 0.03	35.7 ± 6.96	–47.50
DMA_10H	0.20	0.05 ± 0.01	25.9 ± 1.94	–43.52
DMA_20H	0.21	0.07 ± 0.01	34.5 ± 7.39	–41.90
DMA_5T_5H	0.51	0.15 ± 0.03	29.0 ± 3.07	–44.67
DMA_10T_10H	0.62	0.15 ± 0.03	24.4 ± 5.91	–49.63

Thermal properties were investigated via DSC analysis, and glass transition temperature (*t*_g_) was determined. For acrylate-based samples, a clear trend could be observed that the addition of HEMA led to an increase in *t*_g_, while higher amounts of thiol (see Table 3, DA_15T and DA_10T_10H) seemed to have the opposite effect. Similar observations regarding a lower *t*_g_ with higher thiol content were made with PEGDMA-based samples. A previously published study on PEGDMA-thiol hydrogels confirmed a similar value of −49.99 for a PEGDMA hydrogel with added ETTMP.^40^

### Water Uptake Studies

**Figure 5:**
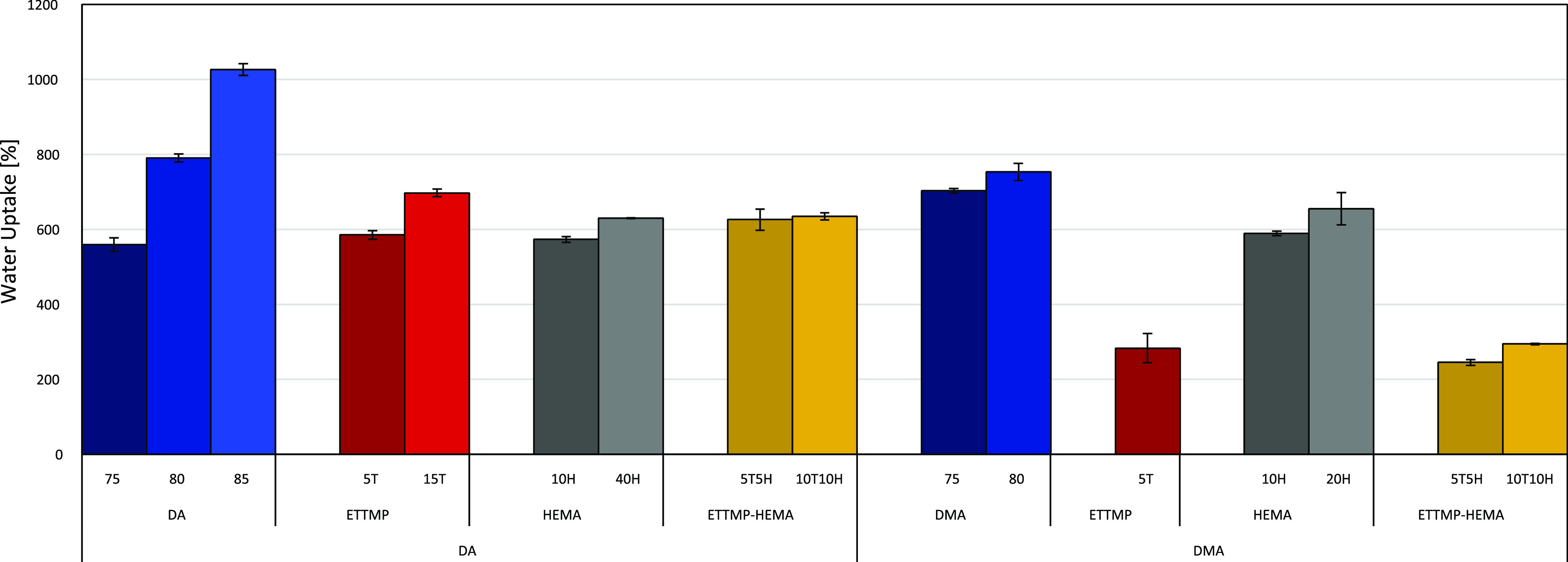
Comparison of the water uptake behavior in PBS at room temperature of the different polyHIPEs after equilibration at 24 h. The data are displayed as mean ± standard deviation from triplicates.

Further, water uptake behavior of the samples in PBS was studied. The experiments were all performed in PBS buffer solution over the course of 96 h to ensure equilibrium. The general observation was that the majority of water uptake happened within the first few minutes after immersion into the solution. The measurements taken after 24 and 96 h did not differ significantly from the ones after 15 min and 1 h (see Supporting Information). Furthermore, it was tested if polyHIPEs with varying porosities and monomer compositions show different uptake behaviors. It was concluded that the most crucial factor for the uptake and swelling ability was the amount of added internal phase, leading to higher porosities. This was especially prominent in the PEGDA-based samples, where the internal phase volume ranged from 75 to 85 vol % (see Figure 5). The weight increased from 560% to over 1000% from 75 to 85 vol % internal phase, respectively. This observation confirmed that macropores and surface area (see Supporting Information) play a more critical role in the water uptake and swelling behavior of the hydrogel than the expanding of chains and hydrogen bonding to the molecules. The addition of ETTMP 1300 and HEMA to acrylate-based HIPE formulations showed no significant impact on the uptake ability. However, the addition of thiol to a methacrylate-formulation drastically decreased the water uptake compared to the other polyHIPEs (see Figure 5: DMA_5T, DMA_5T_5H, and DMA_10T_10H). A reason for this observation could be the relatively low surface area and porosity compared to some of the other samples (see Supporting Information). Even though the void filling of the porous structure appeared to be the primary factor prepared specimens, volume change (swelling) could also be observed for all samples, suggesting a mixed mechanism of pore filling and polymer chain extension. The diameters of the cubic samples were measured dry and again after 24 h of being immersed in water. The increase in volume ranged from 175% (DA_75) to 271% (DA_40H) for all samples.

### Degradation Studies

When biomedical applications are envisioned, degradability is a vital aspect to consider. Our study focused on two different conditions, depending on the intended application of the polyHIPE. Besides PBS buffer at pH = 7.4, an acetate buffer at pH = 4.9 was selected to simulate the more acidic environment of human skin for potential transdermal applications.^41^ Since PEG-based materials typically exhibit low biodegradability, the inclusion of a thiol cross-linker was expected to be beneficial for degradation due to hydrolysis of ester groups present in the thiol molecules, and in this case, also in the PEGDA and PEGDMA molecules. Burke et al. also demonstrated the degradation of PEGDMA-based thiol–ene hydrogels in a recent study.^40^

A comparison between the degradation of polyHIPEs between PBS and acetate buffer clarified that acidic conditions for both PEGDA and PEGDMA systems lead to an overall mass loss increase by the end of the experiment series. Some samples showed a small weight gain within the first one or two weeks, probably due to salt residues from the buffer solution remaining in the porous structure.

Overall, the average weight loss under acidic conditions of PEGDA-based polyHIPEs was 6.6% higher compared to samples immersed in PBS. DA_10H exhibited the highest mass loss over time. For PEGDMA-based polyHIPEs, average weight loss over all samples increased only by 1%. After six weeks, the highest mass loss was observed for DA_5T and DA_10H in acetate buffer with 24 and 27%, respectively. Similar levels of remaining mass within 7 weeks were recorded in a degradation study by Caldwell et al. of thiol–ene polyHIPEs. However, in their study, a 0.1 M NaOH solution was used, making it difficult to compare.^22^ In another study conducted by Johnson et al., polycaprolactone thiol-cross-linked polyHIPEs were fully degraded in 0.01 M NaOH, and the remaining solution was tested for cell toxicity.^42^

Besides recording the weight change of the samples, pH was monitored before being changed. No significant change was recorded each week before the regular exchange of buffer solution. The degradation process for polyHIPEs is not only influenced by the chemical composition of the materials but also of the porous morphology. While samples containing higher amounts of ester linkages are expected to degrade faster, the different resulting morphologies of the materials could lead to an unexpected result.

Overall, the incorporation of at least 10 mol % HEMA leads to higher degradation. PEGDA_5T_5H and PEGDA_10T_10H are an exception of this trend. This could be due to an increasingly complex polymerization process, where acrylates, methacrylates, and thiols with different reactivity undergo polymerization, potentially forming a less degradable polymer network. For PEGDMA_5T_5H and PEGDMA_10T_10H, the molecular structure is different, as only methacrylates and thiols are present in the system. Therefore, different degradation profiles can be expected as well.

**Figure 6:**
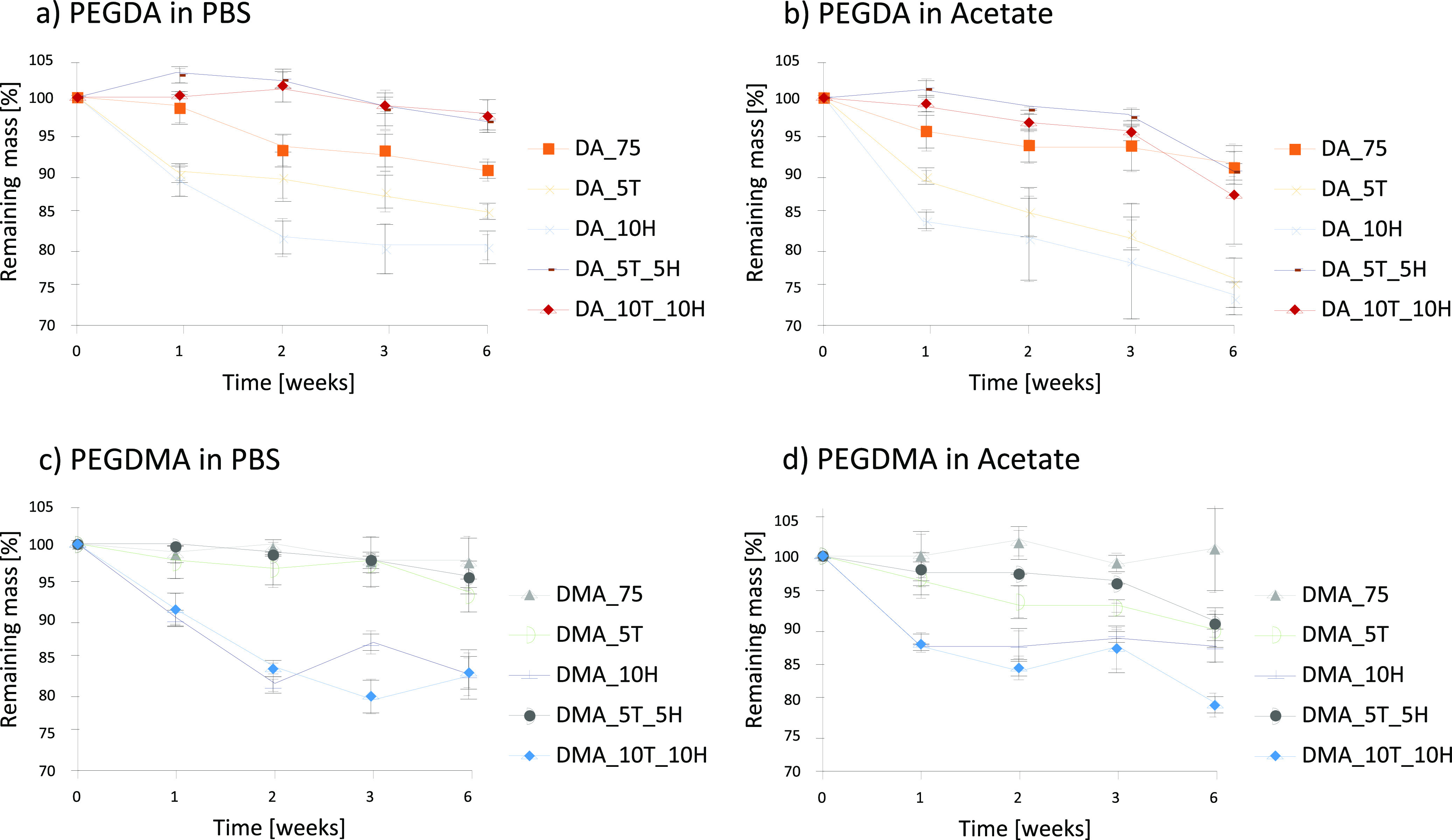
Remaining mass of polyHIPEs in percent over 1, 2, 3, and 6 weeks at 37 °C with different PEGDA-based polyHIPEs in PBS (a) and PEGDMA-based polyHIPEs in PBS (b). The same experiments were performed in acetate buffer for the same PEGDA-based polyHIPEs (c) and PEGDMA-based polyHIPEs (d). The data are displayed as mean ± standard deviation from triplicates.

Due to the demonstrated stability, external (e.g., topical and transdermal) applications can be feasible. If an extension of exposure was to be envisioned, it should be considered that the material degrades over time which is especially crucial for *in vivo* applications (Figure 6).

### Drug Release

Drug loading and release were performed for the samples DA_5T, DA_10T_10H, and DMA_10T_10H. These compositions were selected to examine a difference in release behavior for PEGDA and PEGDMA samples and an effect of the addition of HEMA. Samples consisting of solely acrylate or methacrylate were not selected due to their brittleness. Samples containing HEMA were not chosen for the same reason. A 10% solution of salicylic acid in ethanol was used to load the samples. Salicylic acid was chosen as our model drug due to its widespread use in topical drug delivery application, especially as an agent against acne. The concentrations of salicylic acid in products depend highly on the desired use; for example, too high concentrations of salicylic acid could lead to skin irritation.^43^ Over-the-counter treatments usually contain up to 5% salicylic acid, while prescription treatments might contain more, up to 30%.^40^ Therefore, the loading with a 10% salicylic acid solution was chosen in our study as an intermediate concentration value.

**Figure 7:**
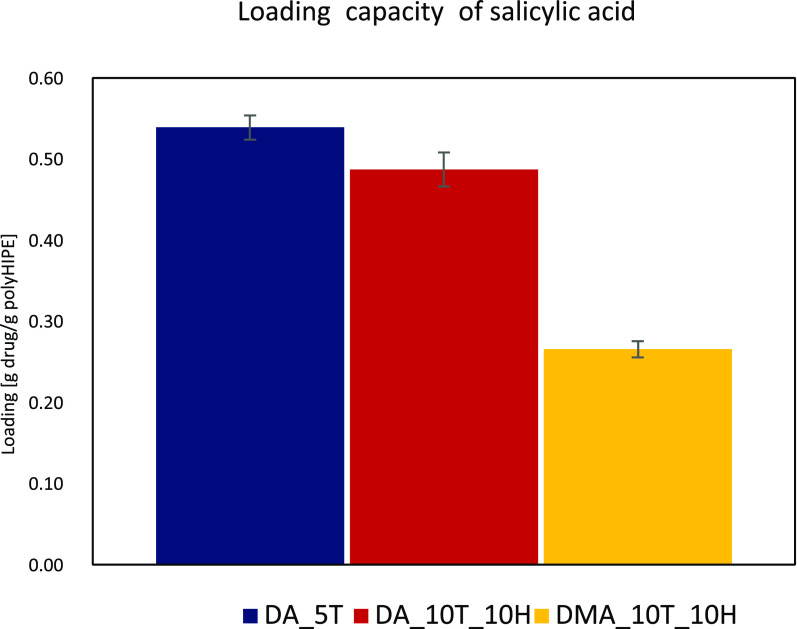
Drug loading behavior of selected polyHIPE samples DA_5T, DA_10T_10H, and DMA_10T_10H.

The samples were dried after being immersed in the drug solution for 24 h. Afterward, the release was performed over 48 h at room temperature in PBS. Upon plotting the received data, a burst release behavior was observed for all samples. This was expected due to the macroporous nature of the synthesized materials. The majority of drug release happened within the first 24 h, with a slowly flattening curve until 48 h passed. The overall release profiles after 24 h were similar for all samples (see Figure 7), leading to the conclusion that chemical composition does not play a vital role in the release.

**Figure 8:**
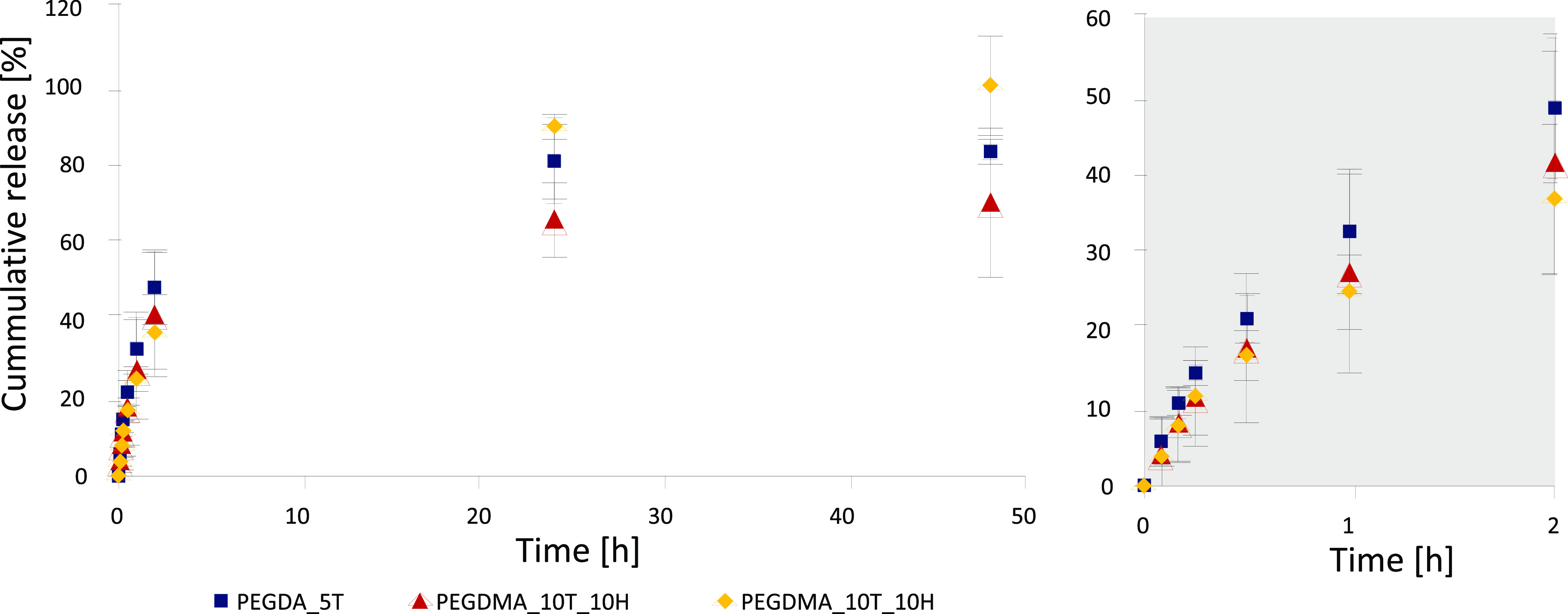
Salicylic acid release profile of the three selected polyHIPE samples DA_5T, DA_10T_10H, and DMA_10T_10H at room temperature in PBS. The release behavior was recorded for 48 h and was plotted as cumulative release in percent. The data are displayed as mean ± standard deviation from triplicates.

After release, the samples were dried and weighed again to determine the amount of remaining loaded drug. However, it has to be noted that the release was performed in PBS, and traces of salt might have a minor influence on the sample weight. For DA_5T, 17% (0.102 g drug/g polyHIPE) of the drug were remaining after the release. DA_10T_10H had the highest amount of salicylic acid left, with 30% (0.142 g drug/g polyHIPE) of the original loaded weight remaining. However, of the three different materials, it reached the highest drug loading per gram polymer (see Figure 7). DMA_10T_10H excreted 100% of the loaded drug within 48 h, but had the least amount of drug loaded by weight of the three different samples as can be seen in Figures 7 and 8.

## Conclusions

Porous polyHIPE hydrogels based on PEGDA or PEGDMA were developed utilizing o/w emulsion templating. For the first time, it was shown that the thiol–ene click reaction can be successfully applied to create polymer networks of with a hydrophilic thiol, ethoxylated trimethylolpropane tri(3-mercaptopropionate), and within an o/w emulsion. At the same time, high porosity, up to 90%, and an open porous interconnected morphology are obtained with direct emulsion templating. Rapid curing and high curing depths via photopolymerization—often a problem with high internal phase emulsions—could be achieved due to the rare phenomenon of emulsion transparency. The hydrophilicity and high porosity of the developed materials facilitate high water uptake. Together with expected biocompatibility and biodegradability, these characteristics are of interest as novel materials for biomedical applications of which drug loading and release of salicylic acid were demonstrated in this study.

## Supporting Information Available

Additional SEM images, water uptake data, tensile tests, and curing by depth images (PDF)

## References

[ref1] Wu J.; Xu F.; Li S.; Ma P.; Zhang X.; Liu Q.; Fu R.; Wu D. Porous Polymers as Multifunctional Material Platforms toward Task-Specific Applications. Adv. Mater. 2019, 31, 1802922 10.1002/adma.201802922. 30345562

[ref2] Hentze H.-P.; Antonietti M. Porous polymers and resins for biotechnological and biomedical applications. Rev. Mol. Biotechnol. 2002, 90, 27–53. 10.1016/S1389-0352(01)00046-0. 12069045

[ref3] Zhang T.; Sanguramath R. A.; Israel S.; Silverstein M. S. Emulsion Templating: Porous Polymers and Beyond. Macromolecules 2019, 52, 5445–5479. 10.1021/acs.macromol.8b02576.

[ref4] Pulko I.; Krajnc P. High Internal Phase Emulsion Templating—A Path To Hierarchically Porous Functional Polymers. Macromol. Rapid Commun. 2012, 33, 1731–1746. 10.1002/marc.201200393. 22907672

[ref5] Kramer S.; Cameron N. R.; Krajnc P. Porous Polymers from High Internal Phase Emulsions as Scaffolds for Biological Applications. Polymers 2021, 13, 1786 10.3390/polym13111786. 34071683 PMC8198890

[ref6] Lee J.-Y.; Tan B.; Cooper A. I. CO2-in-Water Emulsion-Templated Poly(vinyl alcohol) Hydrogels Using Poly(vinyl acetate)-Based Surfactants. Macromolecules 2007, 40, 1955–1961. 10.1021/ma0625197.

[ref7] Luo W.; Zhang S.; Li P.; Xu R.; Zhang Y.; Liang L.; Wood C. D.; Lu Q.; Tan B. Surfactant-free CO2-in-water emulsion-templated poly (vinyl alcohol) (PVA) hydrogels. Polymer 2015, 61, 183–191. 10.1016/j.polymer.2015.02.002.

[ref8] Luo W.; Xu R.; Liu Y.; Hussain I.; Lu Q.; Tan B. Emulsion-templated poly(acrylamide)s by using polyvinyl alcohol (PVA) stabilized CO2-in-water emulsions and their applications in tissue engineering scaffolds. RSC Adv. 2015, 5, 92017–92024. 10.1039/C5RA14345D.

[ref9] Livshin S.; Silverstein M. S. Enhancing hydrophilicity in a hydrophobic porous emulsion-templated polyacrylate. J. Polym. Sci., Part A: Polym. Chem. 2009, 47, 4840–4845. 10.1002/pola.23522.

[ref10] Krajnc P.; Štefanec D.; Pulko I. Acrylic acid “reversed” PolyHIPEs. Macromol. Rapid Commun. 2005, 26, 1289–1293. 10.1002/marc.200500353.

[ref11] Kovačič S.; Štefanec D.; Krajnc P. Highly porous open-cellular monoliths from 2-hydroxyethyl methacrylate based high internal phase emulsions (HIPEs): Preparation and void size tuning. Macromolecules 2007, 40, 8056–8060. 10.1021/ma071380c.

[ref12] Nalawade A. C.; Ghorpade R. V.; Shadbar S.; Qureshi M. S.; Chavan N. N.; Khan A. A.; Ponrathnam S. Inverse high internal phase emulsion polymerization (i-HIPE) of GMMA, HEMA and GDMA for the preparation of superporous hydrogels as a tissue engineering scaffold. J. Mater. Chem. B 2016, 4, 450–460. 10.1039/C5TB01873K. 32263209

[ref13] Golub D.; Krajnc P. Emulsion templated hydrophilic polymethacrylates. Morphological features, water and dye absorption. React. Funct. Polym. 2020, 149, 104515 10.1016/j.reactfunctpolym.2020.104515.

[ref14] Barbetta A.; Dentini M.; Zannoni E. M.; De Stefano M. E. Tailoring the porosity and morphology of gelatin-methacrylate polyHIPE scaffolds for tissue engineering applications. Langmuir 2005, 21, 12333–12341. 10.1021/la0520233. 16343011

[ref15] Barbetta A.; Barigelli E.; Dentini M. Porous alginate hydrogels: synthetic methods for tailoring the porous texture. Biomacromolecules 2009, 10, 2328–2337. 10.1021/bm900517q. 19591464

[ref16] Barbetta A.; Dentini M.; De Vecchis M. S.; Filippini P.; Formisano G.; Caiazza S. Scaffolds Based on Biopolymeric Foams. Adv .Funct .Mater . 2005, 15, 118–124. 10.1002/adfm.200400072.

[ref17] McGann C. L.; Streifel B. C.; Lundin J. G.; Wynne J. H. Multifunctional polyHIPE wound dressings for the treatment of severe limb trauma. Polymer 2017, 126, 408–418. 10.1016/j.polymer.2017.05.067.

[ref18] Kimmins S. D.; Wyman P.; Cameron N. R. Photopolymerised methacrylate-based emulsion-templated porous polymers. Polymers 2012, 72, 947–954. 10.1016/j.reactfunctpolym.2012.06.015.

[ref19] Corti M.; Calleri E.; Perteghella S.; Ferrara A.; Tamma R.; Milanese C.; Mandracchia D.; Brusotti G.; Torre M. L.; Ribatti D.; Auricchio F.; Massolini G.; Tripodo G. Polyacrylate/polyacrylate-PEG biomaterials obtained by high internal phase emulsions (HIPEs) with tailorable drug release and effective mechanical and biological properties. Mater. Sci. Eng. C 2019, 105, 110060 10.1016/j.msec.2019.110060. 31546370

[ref20] Lovelady E.; Kimmins S. D.; Wu J.; Cameron N. R. Preparation of emulsion-templated porous polymers using thiol-ene and thiol-yne chemistry. Polym. Chem. 2011, 2, 559 10.1039/C0PY00374C.

[ref21] Sušec M.; Liska R.; Russmüller G.; Kotek J.; Krajnc P. Microcellular Open Porous Monoliths for Cell Growth by Thiol-Ene Polymerization of Low-Toxicity Monomers in High Internal Phase Emulsions. Macromol. Biosci. 2015, 15, 253–261. 10.1002/mabi.201400219. 25294695

[ref22] Caldwell S.; Johnson D. W.; Didsbury M. P.; Murray B. A.; Wu J. J.; Przyborski S. A.; Cameron N. R. Degradable emulsion-templated scaffolds for tissue engineering from thiol-ene photopolymerisation. Soft Matter 2012, 8, 10344 10.1039/c2sm26250a.

[ref23] Whitely M. E.; Robinson J. L.; Stuebben M. C.; Pearce H. A.; McEnery M. A. P.; Cosgriff-Hernandez E. Prevention of Oxygen Inhibition of PolyHIPE Radical Polymerization Using a Thiol-Based Cross-Linker. ACS Biomater. Sci. Eng. 2017, 3, 409–419. 10.1021/acsbiomaterials.6b00663. 29104917 PMC5663280

[ref24] Greenwald R. B.; Choe Y. H.; McGuire J.; Conover C. D. Effective drug delivery by PEGylated drug conjugates. Adv. Drug Delivery Rev. 2003, 55, 217–250. 10.1016/S0169-409X(02)00180-1. 12564978

[ref25] Fairbanks B. D.; Schwartz M. P.; Bowman C. N.; Anseth K. S. Photoinitiated polymerization of PEG-diacrylate with lithium phenyl-2,4,6-trimethylbenzoylphosphinate: polymerization rate and cytocompatibility. Biomaterials 2009, 30, 6702–6707. 10.1016/j.biomaterials.2009.08.055. 19783300 PMC2896013

[ref26] Khan A. H.; Cook J. K.; Wortmann W. J. III; Kersker N. D.; Rao A.; Pojman J. A.; Melvin A. T. Synthesis and characterization of thiol-acrylate hydrogels using a base-catalyzed Michael addition for 3D cell culture applications. J. Biomed. Mater. Res. 2020, 108, 2294–2307. 10.1002/jbm.b.34565. 31961056

[ref27] Benedikt S.; Wang J.; Markovic M.; Moszner N.; Dietliker K.; Ovsianikov A.; Grützmacher H.; Liska R. Highly efficient water-soluble visible light photoinitiators. J. Polym. Sci., Part A: Polym. Chem. 2016, 54, 473–479. 10.1002/pola.27903.

[ref28] Babak V. G.; Stébé M.-J. Highly Concentrated Emulsions: Physicochemical Principles of Formulation. J. Dispersion Sci. Technol. 2002, 23, 1–22. 10.1080/01932690208984184.

[ref29] Livshin S.; Silverstein M. S. Crystallinity and Cross-Linking in Porous Polymers Synthesized from Long Side Chain Monomers through Emulsion Templating. Macromolecules 2008, 41, 3930–3938. 10.1021/ma800195w. 32907157

[ref30] Gitli T.; Silverstein M. S. Bicontinuous hydrogel-hydrophobic polymer systems through emulsion templated simultaneous polymerizations. Soft Matter 2008, 4, 2475–2485. 10.1039/B809346F.

[ref31] Gurevitch I.; Silverstein M. S. Polymerized pickering HIPEs: effects of synthesis parameters on porous structure. J. Polym. Sci., Part A: Polym. Chem. 2010, 48, 1516–1525. 10.1002/pola.23911.

[ref32] Gurevitch I.; Silverstein M. S. Nanoparticle-Based and Organic-Phase-Based AGET ATRP PolyHIPE Synthesis within Pickering HIPEs and Surfactant-Stabilized HIPEs. Macromolecules 2011, 44, 3398–3409. 10.1021/ma200362u.

[ref33] Cameron N. R.; Sherrington D. C.; Albiston L.; Gregory D. P. Study of the formation of the open-cellular morphology of poly(styrene/divinylbenzene) polyHIPE materials by cryo-SEM. Colloid Polym. Sci. 1996, 274, 592–595. 10.1007/BF00655236.

[ref34] Quell A.; de Bergolis B.; Drenckhan W.; Stubenrauch C. How the Locus of Initiation Influences the Morphology and the Pore Connectivity of a Monodisperse Polymer Foam. Macromolecules 2016, 49, 5059–5067. 10.1021/acs.macromol.6b00494.

[ref35] Robinson J. L.; Moglia R. S.; Stuebben M. C.; McEnery M. A. P.; Cosgriff-Hernandez E. Achieving Interconnected Pore Architecture in Injectable PolyHIPEs for Bone tissue engineering. Tissue Eng., Part A 2014, 20, 1103–1112. 10.1089/ten.TEA.2013.0319. 24124758 PMC3938937

[ref36] Whitely M. E.; Robinson J. L.; Stuebben M. C.; Pearce H. A.; McEnery M. A. P.; Cosgriff-Hernandez E. Prevention of Oxygen Inhibition of PolyHIPE Radical Polymerization Using a Thiol-Based Cross-Linker. ACS Biomater. Sci. Eng. 2017, 3, 409–419. 10.1021/acsbiomaterials.6b00663. 29104917 PMC5663280

[ref37] Zhang T.; Guo Q. Isorefractive high internal phase emulsion organogels for light induced reactions. Chem. Commun. 2016, 52, 4561–4564. 10.1039/C6CC00391E. 26940856

[ref38] Foudazi R. HIPEs to PolyHIPEs. React. Funct. Polym. 2021, 164, 104917 10.1016/j.reactfunctpolym.2021.104917.

[ref39] Bandiera M.; Hamzehlou S.; Ruipérez F.; Aguirre M.; Balk R.; Barandiaran M. J.; Leiza J. R. Copolymerization of (meth)acrylates with vinyl aromatic macromonomers: understanding the mechanism of retardation on the kinetics with acrylates. Polym. Chem. 2019, 10, 1769–1779. 10.1039/C9PY00062C.

[ref40] Burke G.; Cao Z.; Devine D. M.; Major I. Preparation of Biodegradable Polyethylene Glycol Dimethacrylate Hydrogels via Thiol-ene Chemistry. Polymers 2019, 11, 1339 10.3390/polym11081339. PMC6722562 31412552

[ref41] Lambers H.; Piessens S.; Bloem A.; Pronk H.; Finkel P. Natural skin surface pH is on average below 5, which is beneficial for its resident flora. Int. J. Cosmet. Sci. 2006, 28, 359–370. 10.1111/j.1467-2494.2006.00344.x. 18489300

[ref42] Johnson D. W.; Langford C. R.; Didsbury M. P.; Lipp B.; Przyborski S. A.; Cameron N. R. Fully biodegradable and biocompatible emulsion templated polymer scaffolds by thiol-acrylate polymerization of polycaprolactone macromonomers. Polym. Chem. 2015, 6, 7256–7263. 10.1039/C5PY00721F.

[ref43] Decker A.; Graber E. M. Over-the-counter Acne Treatments: A Review. J. Clin. Aesthet. Dermatol. 2012, 5, 32–40. 22808307 PMC3366450

