# Supplementary material for: Thiol–Ene Cross-linking of Poly(ethylene glycol) within High Internal Phase Emulsions: Degradable Hydrophilic PolyHIPEs for Controlled Drug Release
Source: Macromolecules. 2021 Nov 8;54(22):10370–80. doi: 10.1021/acs.macromol.1c01240 (PMC8619294; doi:10.1021/acs.macromol.1c01240)
Supplement: Supplementary file 1 — ma1c01240_si_001.pdf [file ma1c01240_si_001.pdf]

Thiol-ene crosslinking of poly(ethylene glycol) within high internal phase emulsions: degradable hydrophilic polyHIPEs for controlled drug release

*Viola Hobiger[a], Anna Zahoranova[b], Stefan Baudis[b], Robert Liska[b], Peter Krajnc[a]**

[a]PolyOrgLab, Faculty of Chemistry and Chemical Engineering, University of Maribor, Smetanova 17, Maribor, Slovenia

[b]Institute of Applied Synthetic Chemistry, Vienna University of Technology, Getreidemarkt 9/163, Vienna, Austria

peter.krajnc@um.si

Supporting Information

Sample	BET surface area [m²/g]	Skeletal density [g/cm³]	Theoretical water uptake[a] [%]	Water uptake [%]
**PEGDA_75**	5.6	1.39	647	559
**PEGDA_80**	7.5	1.61	559	790
**PEGDA_85**	8.3	1.65	539	1026
**PEGDA_5T**	5.0	1.32	652	585
**PEGDA_15T**	0.9	1.22	500	697
**PEGDA_10H**	4.8	1.41	610	573
**PEGDA_40H**	5.5	1.29	666	630
**PEGDA_5T_5H**	3.4	1.28	671	626
**PEGDA_10T_10H**	3.1	1.24	589	635
**PEGDMA_75**	7.0	1.51	563	703
**PEGDMA_80**	7.1	1.60	563	753
**PEGDMA_5T**	4.1	1.39	554	283
**PEGDMA_10H**	5.0	1.41	582	589
**PEGDMA_20H**	4.7	1.28	601	655
**PEGDMA_5T_5H**	2.2	1.22	615	245
**PEGDMA_10T_10H**	2.2	1.24	387	294

Table S 1: BET surface areas, skeletal density and water uptake in numbers of all synthesized samples. [a] Theoretical water uptake without swelling calculated from measured porosity and skeletal density.

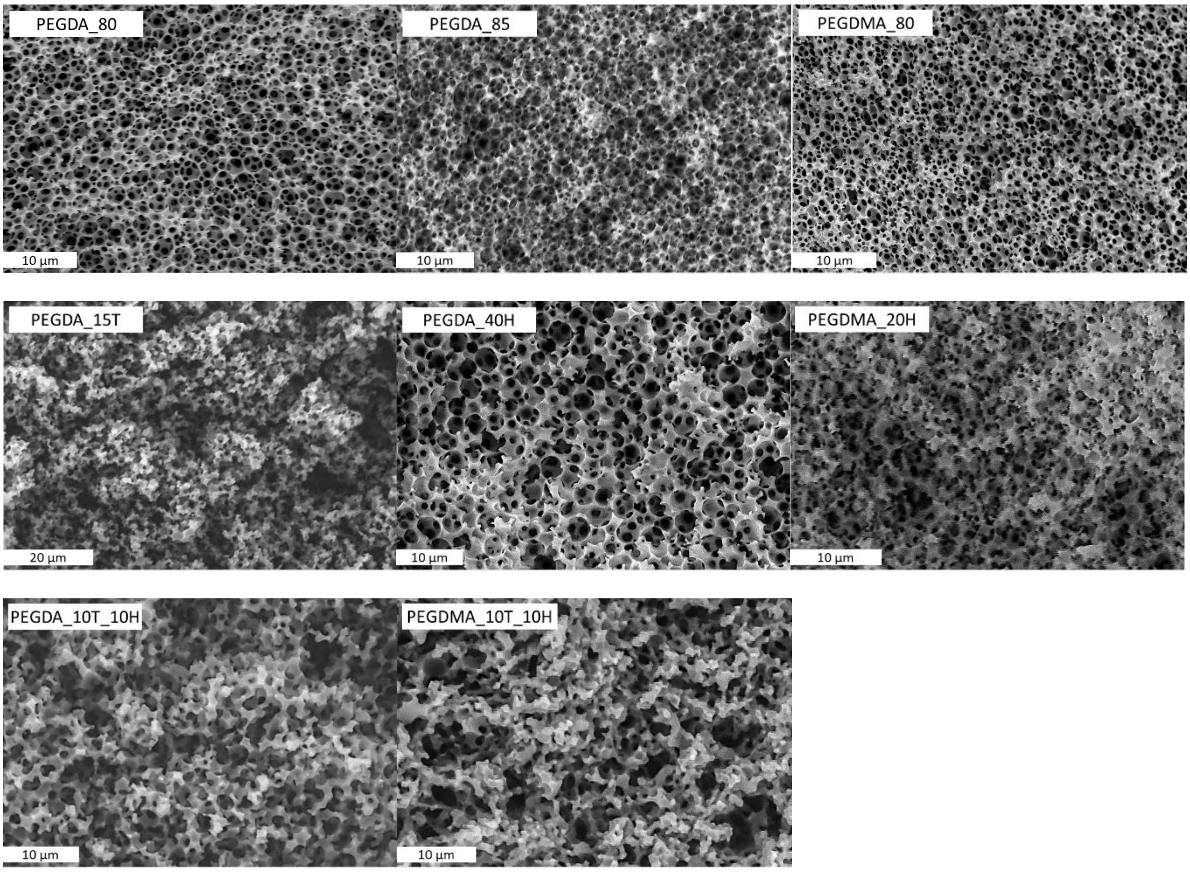

Figure S 2: SEM micrographs of selected PEDGA and PEGDMA polyHIPE samples.

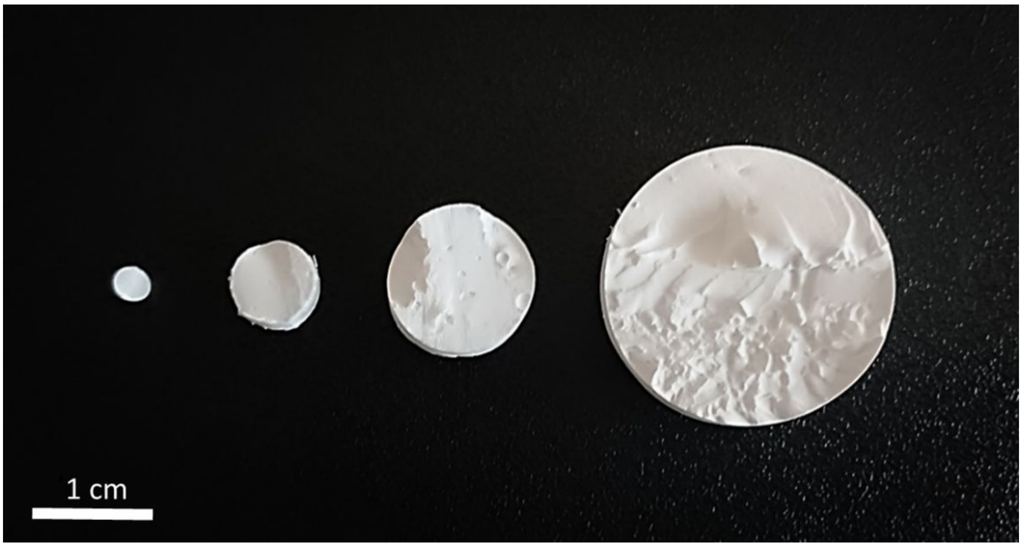

Figure S 3: Shows different curing depths of a DMA_75 sample. Curing of these specimen was performed in a self-built UV-chamber lined with aluminium foil. A UV-lamp (Vilber, VL-6.LC, wavelength: 365 nm, power: 1 x 6 W) was used and samples were irradiated for two hours.

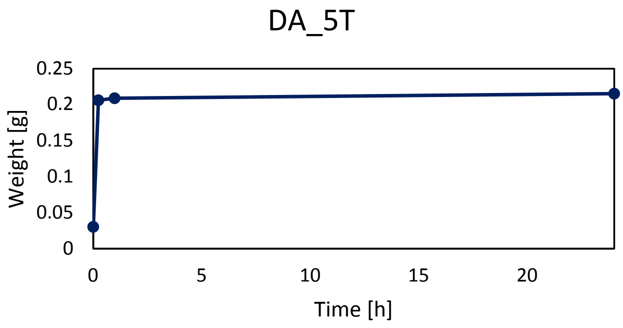

Figure S 4: Exemplary water uptake behavior of the selected sample, DA_75.

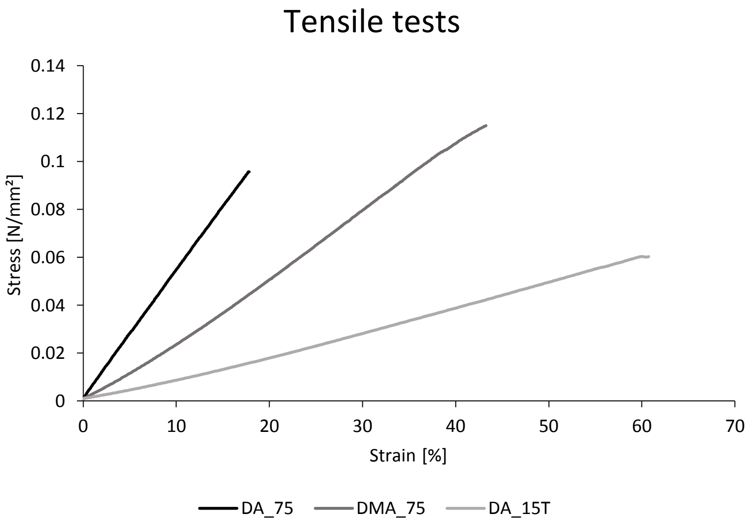

Figure S 5: Stress strain curves of three selected samples, DA_75, DMA_75 and DA_15T.
